# Supplementary material for: The Systems Biology Research Tool: evolvable open-source software
Source: BMC Syst Biol. 2008 Jun 29;2:55. doi: 10.1186/1752-0509-2-55 (PMC2446383; doi:10.1186/1752-0509-2-55)
Supplement: Additional file 1 — SBRT Archive. An archive of the current version of the Systems Biology Research Tool. [file 1752-0509-2-55-S1.zip › sbrt-1.4.0/doc/developers_guide/api/sbrt/shell/mng/class-use/KernelProcessManager.html]

Uses of Interface sbrt.shell.mng.KernelProcessManager


|  |  |  |
| --- | --- | --- |
| **Overview** \| **Package** \| **Class** \| **Use** \| **Tree** \| **Deprecated** \| **Index** \| **Help** | |  |
| PREV   NEXT | **FRAMES**    **NO FRAMES**     **All Classes** |  |


---


## **Uses of Interface sbrt.shell.mng.KernelProcessManager**

| Packages that use KernelProcessManager | |
| --- | --- |
| **sbrt.shell.mng.algebra** | Provides classes for creating algebra process managers. |
| **sbrt.shell.mng.comb** | Provides classes for creating combinatorics process managers. |
| **sbrt.shell.mng.fba** | Provides classes and interfaces for creating Flux Balance Analysis process managers. |
| **sbrt.shell.mng.geom** | Provides classes and for creating geometry process managers. |
| **sbrt.shell.mng.graph\_theory** | Provides classes and for creating graph theoretical process managers. |
| **sbrt.shell.mng.stat** | Provides classes and interfaces for creating statistical process managers. |
| **sbrt.shell.mng.utilities** | Provides classes and interfaces for creating mathematical process managers. |

Uses of KernelProcessManager in sbrt.shell.mng.algebra

| Classes in sbrt.shell.mng.algebra that implement KernelProcessManager | |
| --- | --- |
| `class` | `LinearSolverManager`             This class is used to manage linear system solvers. |

Uses of KernelProcessManager in sbrt.shell.mng.comb

| Classes in sbrt.shell.mng.comb that implement KernelProcessManager | |
| --- | --- |
| `class` | `SingleElementUnionManager`             This class is used to manage single-element union computers. |
| `class` | `StrictSingleElementUnionManager`             This class is used to manage single-element union computers. |

Uses of KernelProcessManager in sbrt.shell.mng.fba

| Classes in sbrt.shell.mng.fba that implement KernelProcessManager | |
| --- | --- |
| `class` | `CatalystDeletionManager`             This class is used to manage FBA catalyst deletion experiments. |
| `class` | `CdHarFluxVectorManager`             This class is used to manage FBA coordinate direction hit-and-run random flux vector experiments. |
| `class` | `CvCatalystDeletionManager`             This class is used to manage FBA constraint variation-catalyst deletion experiments. |
| `class` | `CvFbaOptManager`             This class is used to manage constraint variation-FBA optimization processes. |
| `class` | `CvMsFluxIntervalManager`             This class is used to manage constraint variation-Mahadevan-Schilling flux interval experiments. |
| `class` | `CvObjectiveFunctionManager`             This class is used to manage FBA constraint variation-objective function experiments. |
| `class` | `CvRevRxnFluxIntervalManager`             This class is used to manage constraint variation-constrained reverse reaction flux interval experiments. |
| `class` | `CvRxnDeletionManager`             This class is used to manage FBA constraint variation-reaction deletion experiments. |
| `class` | `CvSimpleFluxIntervalManager`             This class is used to manage constraint variation-simple flux interval experiments. |
| `class` | `ExtremeCurrentManager`             This class is used to manage FBA extreme current experiments. |
| `class` | `FbaManager`             This abstract class provides a foundation for Flux Balance Analysis managers. |
| `class` | `FbaOptManager`             This class is used to manage basic FBA optimization experiments. |
| `class` | `FbaSolverManager`             This class is used to manage FBA solver processes. |
| `class` | `FluxActivityManager`             This class is used to manage flux activity experiments. |
| `class` | `FluxCapManager`             This class is used to manage flux cap experiments. |
| `class` | `FluxPlasticityManager`             This class is used to manage flux plasticity experiments. |
| `class` | `InitialPointManager`             This class is used to manage initial point experiments. |
| `class` | `IntervalVariationManager`             This abstract class provides utitlity functions for managing flux interval variation experiments. |
| `class` | `MsFluxIntervalManager`             This class is used to manage Mahadevan-Schilling flux interval experiments. |
| `class` | `MsFluxomeReducerManager`             This class is used to manage the MS fluxome reduction algorithm. |
| `class` | `NetworkInfoManager`             This class is used to manage fluxome information experiments. |
| `class` | `ObjectiveFunctionManager`             This class is used to manage objective function experiments. |
| `class` | `RandomConstraintsManager`             This class is used to manage random constraints generators. |
| `class` | `RandomEnvManager`             This class is used to manage random environment generators. |
| `class` | `RandomFluxVectorManager`             This abstract class provides a foundation for random flux vector managers. |
| `class` | `RandomObjectiveManager`             This class is used to manage random objective function generators. |
| `class` | `ReverseRxnFluxIntervalManager`             This class is used to manage constrained-reverse reaction flux interval experiments. |
| `class` | `RxnDeletionManager`             This class is used to manage FBA reaction deletion experiments. |
| `class` | `SimpleFluxIntervalManager`             This class is used to manage simple flux interval experiments. |
| `class` | `SlpCycleIdManager`             This class is used to manage steady state cycle identification. |
| `class` | `StoichVariationManager`             This class is used to manage stoichiometry variation-FBA optimization processes. |
| `class` | `SvCdHarFluxVectorManager`             This class is used to manage flux space variation, coordinate direction hit-and-run random flux vector experiments. |
| `class` | `SvInitialPointManager`             This class is used to manage flux space variation-initial point experiments. |
| `class` | `WwFluxomeReducerManager`             This class is used to manage the WW fluxome reduction algorithm. |

Uses of KernelProcessManager in sbrt.shell.mng.geom

| Classes in sbrt.shell.mng.geom that implement KernelProcessManager | |
| --- | --- |
| `class` | `CdHarManager`             This class is used to manage coordinate direction hit-and-run algorithms. |

Uses of KernelProcessManager in sbrt.shell.mng.graph\_theory

| Classes in sbrt.shell.mng.graph\_theory that implement KernelProcessManager | |
| --- | --- |
| `class` | `CycleIdManager`             This class is used to manage simple cycle identification experiments. |
| `class` | `PathIdManager`             This class is used to manage simple path identification experiments. |

Uses of KernelProcessManager in sbrt.shell.mng.stat

| Classes in sbrt.shell.mng.stat that implement KernelProcessManager | |
| --- | --- |
| `class` | `CorrelationManager`             This class is used to manage correlation processes. |
| `class` | `KendallTauManager`             This class is used to manage correlation process whose correlation computers are of type `KendallTau`. |
| `class` | `MannWhitneyUManager`             This class is used to manage Mann-Whitney tests. |

Uses of KernelProcessManager in sbrt.shell.mng.utilities

| Classes in sbrt.shell.mng.utilities that implement KernelProcessManager | |
| --- | --- |
| `class` | `IntervalComparisonManager`             This class is used to compare the intervals contained in separate files. |
| `class` | `VariableParticipationManager`             This class is used to manage variable participation experiments. |
| `class` | `VectorComparisonManager`             This class is used to compare the intervals contained in separate files. |

---


|  |  |  |
| --- | --- | --- |
| **Overview** \| **Package** \| **Class** \| **Use** \| **Tree** \| **Deprecated** \| **Index** \| **Help** | |  |
| PREV   NEXT | **FRAMES**    **NO FRAMES**     **All Classes** |  |


---
